# In Vivo Morphological Features of Human Lumbar Discs

**DOI:** 10.1097/MD.0000000000000333

**Published:** 2014-12-02

**Authors:** Weiye Zhong, Sean J. Driscoll, Minfei Wu, Shaobai Wang, Zhan Liu, Thomas D. Cha, Kirkham B. Wood, Guoan Li

**Affiliations:** From the Bioengineering Laboratory (WZ, SJD, MW, SW, ZL, TDC, KBW, GL), Department of Orthopedic Surgery, Harvard Medical School/Massachusetts General Hospital, Boston, MA; Department of Spinal Surgery (WZ), Second Xiangya Hospital and Central South University, Changsha, Hunan; and Department of Orthopedics (MW), China-Japan Union Hospital of Jilin University, Jilin, P.R. China.

## Abstract

Recent biomechanics studies have revealed distinct kinematic behavior of different lumbar segments. The mechanisms behind these segment-specific biomechanical features are unknown. This study investigated the in vivo geometric characteristics of human lumbar intervertebral discs.

Magnetic resonance images of the lumbar spine of 41 young Chinese individuals were acquired. Disc geometry in the sagittal plane was measured for each subject, including the dimensions of the discs, nucleus pulposus (NP), and annulus fibrosus (AF). Segmental lordosis was also measured using the Cobb method.

In general, the disc length increased from upper to lower lumbar levels, except that the L4/5 and L5/S1 discs had similar lengths. The L4/5 NP had a height of 8.6 ± 1.3 mm, which was significantly higher than all other levels (*P* < 0.05). The L5/S1 NP had a length of 21.6 ± 3.1 mm, which was significantly longer than all other levels (*P* < 0.05). At L4/5, the NP occupied 64.0% of the disc length, which was significantly less than the NP of the L5/S1 segment (72.4%) (*P* < 0.05). The anterior AF occupied 20.5% of the L4/5 disc length, which was significantly greater than that of the posterior AF (15.6%) (*P* < 0.05). At the L5/S1 segment, the anterior and posterior AFs were similar in length (14.1% and 13.6% of the disc, respectively). The height to length (H/L) ratio of the L4/5 NP was 0.45 ± 0.06, which was significantly greater than all other segments (*P* < 0.05). There was no correlation between the NP H/L ratio and lordosis.

Although the lengths of the lower lumbar discs were similar, the geometry of the AF and NP showed segment-dependent properties. These data may provide insight into the understanding of segment-specific biomechanics in the lower lumbar spine. The data could also provide baseline knowledge for the development of segment-specific surgical treatments of lumbar diseases.

## INTRODUCTION

Lumbar degenerative disc diseases (DDDs) are often found at the lower lumbar levels.^1–3^ Epidemiology studies have suggested a segment-dependent discrepancy in the development of certain pathologies. For example, lumbar disc herniation is found most often at the L5/S1 segment,^1–6^ whereas lumbar degenerative spondylolisthesis is found most often at L4/5.^7–10^ Further, segment-dependent clinical outcomes following surgical treatment of L4/5 or L5/S1 have also been reported. Microdiscectomy of L5/S1 has shown superior clinical outcomes compared with the L4/5,^11^ whereas total lumbar disc replacement at L4/5 has shown superior clinical outcomes compared with the L5/S1.^12^ However, knowledge on segment-specific physiology and clinical outcomes is limited.

There are various potential factors that could influence segment-dependent physiological function of the lumbar spine, including the surrounding muscles, ligaments, and vertebral geometries.^13,14^ However, most biomechanics studies of the lumbar spine make no distinction between different segmental levels. For example, previous reports on pressure measurement and kinematics of the nucleus pulposus (NP) did not provide level-specific analysis.^15–17^ Recent kinematic studies revealed distinct motion characteristics between the L4/5 and L5/S1 segments.^18–20^ Knowledge on the mechanisms behind these segment-dependent pathologies and motion characters could be instrumental for the development of segment-dependent surgeries to treat lumbar diseases.

Therefore, the objective of this study was to investigate the in vivo morphology of lumbar intervertebral discs (IVDs) at different segment levels using sagittal plane magnetic resonance (MR) images. Specifically, the dimensions of the NP and anterior/posterior annulus fibrosus (AF) were compared. The height to length (H/L) ratio of the NP was also calculated to investigate NP shapes. We hypothesize that the IVD has distinct geometric features at different levels of the lumbar spine.

## MATERIALS AND METHODS

MR images of the lumbar spine were obtained from 41 young Chinese subjects (18 men and 23 women, aged 20–35 years) with institutional review board approval of the authors’ institution. Each subject was scanned using a 3.0 Tesla scanner (Achieva X-series; Philips, Eindhoven, The Netherlands) in a nonweight-bearing supine position with a spine surface coil and a T2-weighted fat-suppressed 3D SPGR sequence (TE = 120 ms, TR = 3100 ms, FOV = 300 mm). Parallel sagittal images with a thickness of 4.0 mm, gap of 1.0 mm, and resolution of 256 × 256 pixels were obtained. The lumbar discs were examined using Pfirrmann classification.^21^ Any of the lumbar discs of Pfirrmann grade III–V was used for exclusion of the subject from the study.

**FIGURE 1:**
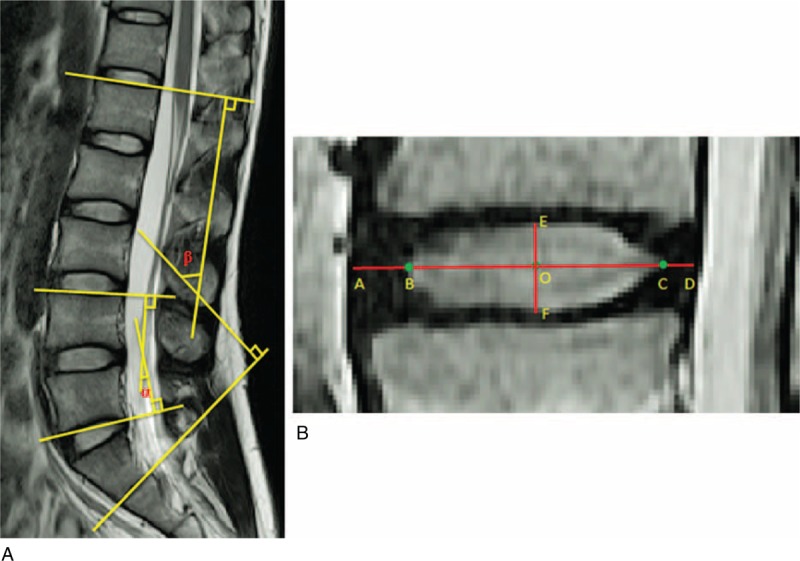
(A) Segmental lordosis (α) and L1-S1 lordosis (β) measurements were made using the Cobb method. Segmental lordosis was defined as the angle subtended by the superior endplate line of the superior vertebrae and the inferior endplate line of the inferior vertebrae. L1-S1 lordosis was defined as the angle subtended by the superior endplate line of L1 and inferior endplate line of S1. (B) Disc geometry was measured from the sagittal plane. AB = anterior AF, AD = disc length, BC = NP length, CD = posterior AF, EF = NP height, O = midpoint of BC. AF = annulus fibrosus, NP = nucleus pulposus.

Geometric measurements for each subject were taken from the T2-weighted midsagittal images using a commercial software program (Rhinoceros; Robert McNeel & Associates, Seattle, WA) (Figure 1). The length of the NP was defined as a midline drawn in the anterior–posterior direction between the margins of the NP. The height of the NP was defined by a perpendicular line through the midpoint of the length, between the inferior and superior margins of the NP. These measurements also allowed for the H/L ratio of the NP to be calculated. The line that defined the NP length was then extended to the anterior and posterior margins of the IVD. The distance of anterior extension was defined as the anterior AF length. The distance of posterior extension was defined as the posterior AF length. The total length of the extended line was defined as the disc length. Finally, segmental lordosis and L1-S1 lordosis were measured using the Cobb method.^22^

A repeated measure of 2-way analysis of variance was used to compare the disc length, NP length, AF length, NP height, H/L ratio, and segmental Cobb angle across lumbar levels. A statistical difference was achieved when *P* < 0.05. A Newman–Keuls post hoc test was performed when a statistically significant difference was detected. NP H/L ratio and lordosis were also correlated using Pearson tests. The statistical analysis was done using the STATISTICA software version 12 (Statsoft Inc, Tulsa, OK).

## RESULTS

**FIGURE 2:**
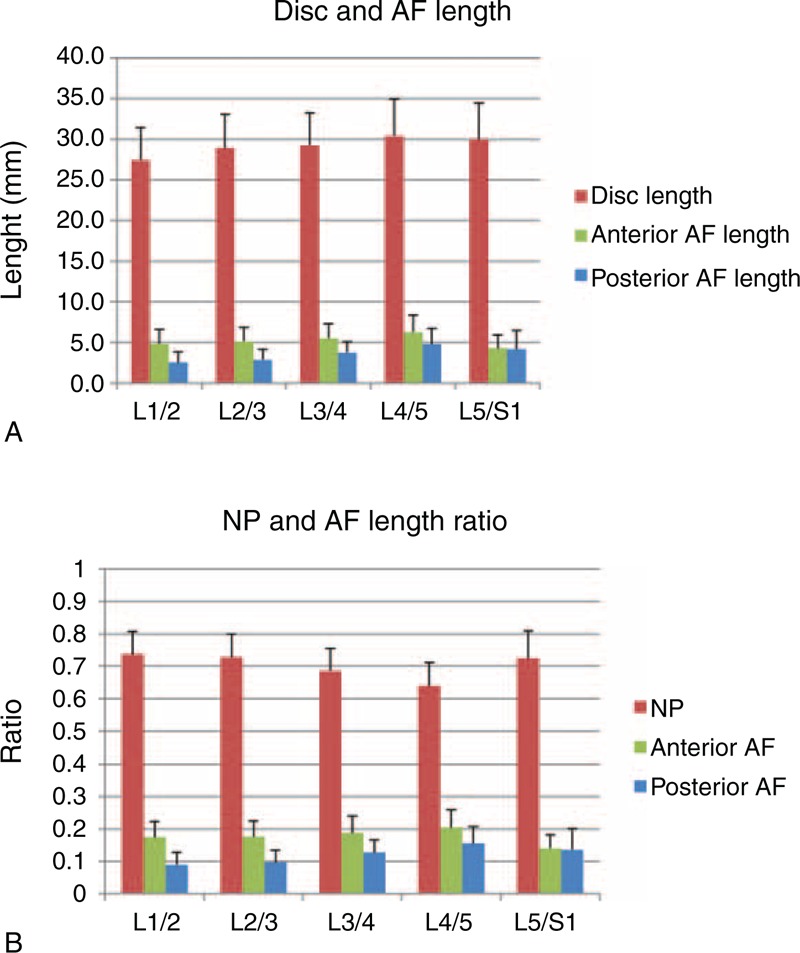
(A) Disc, anterior AF, and posterior AF lengths. Disc length increased from L1/2 to L4/5, with L4/5 and L5/S1 having similar lengths. Anterior AF was significantly longer than posterior AF at all levels except L5/S1. (B) NP, anterior AF, and posterior AF lengths to disc length ratios. NP length ratio significantly decreased from L2/3 to L4/5, before statistically increasing at L5/S1. Anterior AF represented a significantly greater percentage of disc length than posterior AF at all levels except L5/S1. AF = annulus fibrosus, NP = nucleus pulposus.

**FIGURE 3:**
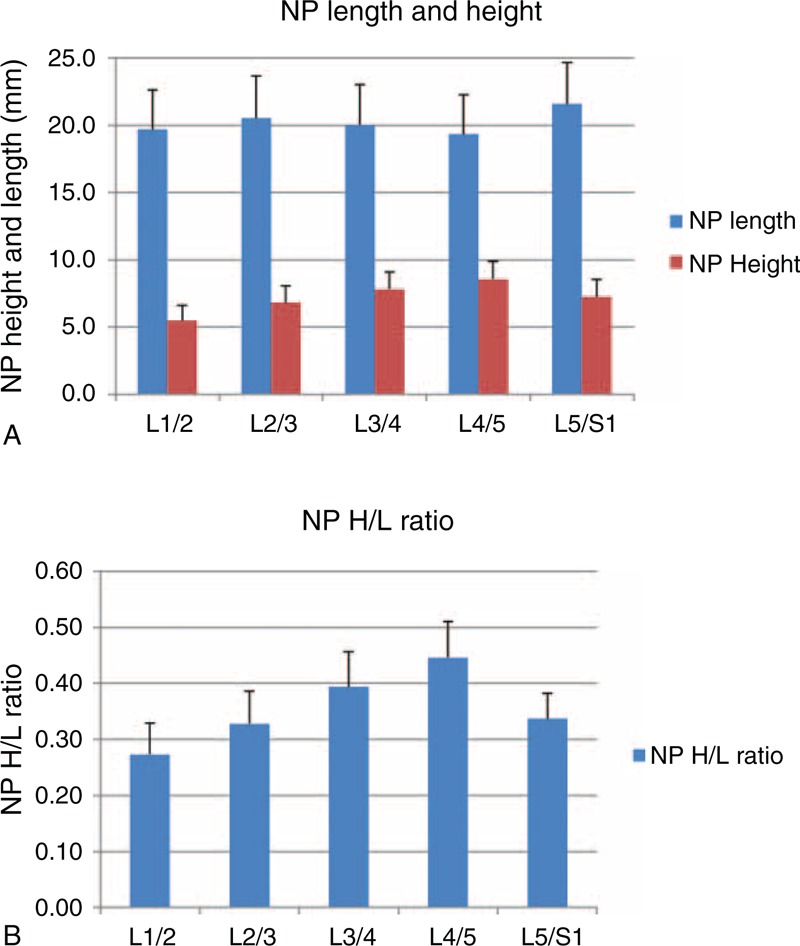
(A) NP length and height. NP length significantly decreased from L2/3 to L4/5, before significantly increasing at L5/S1. NP height significantly increased at each level from L1/2 to L4/5, before significantly decreasing at L5/S1. NP height was the greatest at the L4/5 segment, whereas NP length was greatest at L5/S1. (B) NP H/L ratio. The NP H/L ratio significantly increased from L1/2 to L4/5, before significantly decreasing at L5/S1. The L4/5 NP H/L ratio was significantly greater than all other segments. H/L = height to length, NP = nucleus pulposus.

The length of the discs gradually increased from upper to lower levels (Figure 2a). However, the L4/5 and L5/S1 levels had similar lengths (30.4 ± 4.5 mm and 30.0 ± 4.4 mm, respectively). The NP length of the L5/S1 segment (21.6 ± 3.1 mm) was significantly longer than all other levels (*P* < 0.05). L4/5 had the shortest NP length (19.3 ± 2.9 mm) (Figure 3a). The anterior AF occupied 20.5% of the L4/5 disc length, which was significantly greater than that of the posterior AF (15.6%) (*P* < 0.05) (Figure 2b). At the L5/S1 segment, the anterior and posterior AFs were similar in length (4.3 ± 1.6 mm and 4.1 ± 2.3 mm, respectively), representing 14.1% and 13.6% of the disc, respectively. In all levels, the NP length was significantly longer than both anterior and posterior AF lengths (*P* < 0.05) (Figures 2a and 3a).

The NP height gradually increased from L1/2 (5.5 ± 1.1 mm) to L4/5 (8.6 ± 1.3 mm), and then decreased at L5/S1 (7.3 ± 1.3 mm) (Figure 3a). The L4/5 NP height was significantly greater than all other NPs (*P* < 0.05). The NP H/L ratio showed a similar trend to NP height, gradually increasing from L1/2 (0.27 ± 0.06) to L4/5 (0.45 ± 0.06) before decreasing at L5/S1 (0.34 ± 0.05) (Figure 3b). The NP H/L ratio at L4/5 was significantly greater than all other lumbar segments (*P* < 0.05).

The overall lordosis of L1-S1 was 55.6 ± 9.0°. Segmental lordosis was similar at L1/2 and L2/3 (4.9 ± 3.2° and 4.2 ± 3.1°, respectively) before significantly increasing at each subsequent lower lumbar level (Table 1). The L5/S1 segment had a segmental lordosis of 37.5 ± 5.7°, which was significantly greater than all other levels (*P* < 0.05). There was no correlation between the NP H/L ratio and segmental lordosis (*R*^2^ < 0.26).

**TABLE 1:**
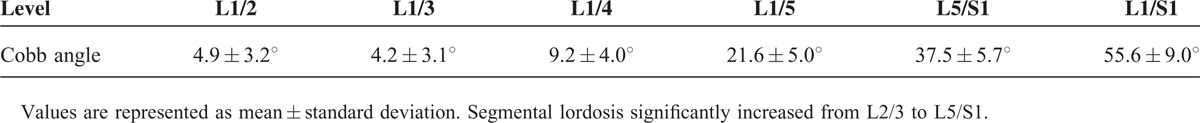
Segmental and L1-S1 Lordosis

## DISCUSSION

This study investigated the in vivo morphology of lumbar IVDs using MR images of 41 healthy human subjects. The data showed segment-dependent geometric features of the lumbar IVD. Notably, NP height was the greatest at the L4/5 segment and NP length was the greatest at the L5/S1 segment. Additionally, the anterior AF was longer than the posterior AF at all lumbar segments except L5/S1, where both the lengths were similar. The data proved our hypothesis that the IVD has distinct geometric features at different levels of the lumbar spine.

Numerous studies have investigated the biomechanics of the lumbar IVD using in vitro or in vivo experimental setups.^23–29^ In vitro cadaveric tests have used various segments to examine the biomechanical responses of the disc to external loads.^23–26^ Finite element studies have also simulated loading of the lumbar spine at multiple levels.^23,28,29^ Although these studies have greatly improved our understanding of disc loading, few studies have specifically compared the biomechanics of different levels.

Ranu^15^ investigated the pressure–volume relation within the NP of intact human cadaveric lumbar discs using a miniature strain gauge. NP pressure was found to rise rapidly with continuous saline infusion. Krag^24^ and Seroussi^25^ examined NP displacement by implanting metal beads into cadaveric IVDs and found that the NP displaced anteriorly in extension and posteriorly in flexion. Schnebel et al^30^ used discography to study the in vivo movement of the lumbar NP in response to flexion and extension. They found movement in the anterior and posterior portions of the disc to be the highest in the L4/5 and L5/S1 NPs, respectively. Subsequent in vivo kinematic studies of the NP during flexion and extension using magnetic resonance imaging (MRI) techniques demonstrated that the upper lumbar levels had more movement than the lower levels in the anterior portion of the disc, whereas the lower levels had more movement than the upper levels in the posterior portion of the disc.^16,17,31–33^ Recently, Alexander^34^ and Nazari^35^ used an open-MRI technique to investigate functional weight-bearing positions of the body (sitting and standing), and found that the in vivo deformation and migration of the lower lumbar NPs (L4/5 and L5/S1 levels) were more affected by position than the upper lumbar NPs. Most of these studies consider the NP to be a hydraulic cushion within the IVD, acting only to distribute stress evenly between vertebrae.^36^ Few reports have discussed segment-dependent geometrical features of the IVD.

Degenerative pathologies of the lumbar spine are often found at the lower levels of L4/5 and L5/S1.^1–3^ Numerous studies have attempted to elucidate factors for DDD such as age, body weight, physical job activities, and vertebral motion features.^2,18^ Although lumbar disc herniation has been reported more often at L5/S1^1–6^ and lumbar degenerative spondylolisthesis more often at L4/5,^7–10^ surgical treatments of L4/5 or L5/S1 have also reported segment-dependent clinical outcomes.^11,12^ Recent in vivo kinematic studies have revealed distinct motion characteristics at the L4/5 and L5/S1 segments.^18–20^ However, few data has been reported on the segment-specific morphology of lumbar IVDs and its potential relationship to these segment-dependent features.

In our study, the L4/5 NP was found to have the greatest height and smallest length among all segments. Additionally, the anterior AF was significantly thicker than the posterior AF at the L4/5 level. The L5/S1 NP was significantly longer than all other levels, with its anterior and posterior AFs being similar in thickness. Our data also demonstrated segment-dependent NP shapes. The NP H/L ratio gradually increased from L1/2 to L4/5 and then decreased at L5/S1. The L4/5 NP showed the largest H/L ratio and was significantly greater than all other levels. There was no correlation between the NP H/L ratio and segmental lordosis in this group of subjects. Further study is warranted to examine how these geometric features may correlate with the distinct kinematic features of the lumbar spine.

Several limitations of this study should be considered. First, it was limited to investigation of IVD morphology in the sagittal plane. Three-dimensional analysis would potentially provide an even better understanding of IVD geometry. Another limitation of this study is the relatively narrow age range of subjects. Future studies should consider age, sex, ethnicity, and body height/weight as study variables. Finally, IVD morphology was studied in a supine position under nonweight-bearing conditions. Future studies should investigate lumbar IVD geometry under various physiological loading conditions. Despite the above limitations, this study represents the first in vivo measurement and analysis of segment-dependent lumbar IVD morphology.

In conclusion, this study investigated human lumbar IVD geometry using sagittal plane MR images and found segment-dependent properties. Notably, the NP of the L4/5 segment had the greatest height, whereas the NP of L5/S1 had the greatest length. These baseline data may provide insight into the understanding of segment-specific pathology and biomechanics in the lower lumbar spine. These data may also be instrumental for the development of segment-specific surgical treatments that are aimed to restore native spine function.
